# Comparative Evaluation of Hydrothermally Produced Rice Starch–Phenolic Complexes: Contributions of Phenolic Type, Plasma-Activated Water, and Ultrasonication

**DOI:** 10.3390/foods11233826

**Published:** 2022-11-27

**Authors:** Paramee Chumsri, Worawan Panpipat, Ling-Zhi Cheong, Mudtorlep Nisoa, Manat Chaijan

**Affiliations:** 1 Food Technology and Innovation Research Center of Excellence, School of Agricultural Technology and Food Industry, Walailak University, Nakhon Si Thammarat 80160, Thailand; 2 Zhejiang-Malaysia Joint Research Laboratory for Agricultural Product Processing and Nutrition, College of Food and Pharmaceutical Science, Ningbo University, Ningbo 315211, China; 3 School of Science, Walailak University, Nakhon Si Thammarat 80160, Thailand

**Keywords:** starch–phenolic complex, phenolics, plasma-activated water, ultrasonication, resistant starch, digestibility, antioxidant, rice starch, functional ingredient

## Abstract

A thorough investigation of the viability of rice starch conjugation with three different phenolic compounds—gallic acid, sinapic acid, and crude Mon-pu (*Glochidion wallichianum* Muell Arg) (MP) extract—was conducted using a variety of developed methods which modified the techno-functionality and digestibility of the end product. With and without the aid of ultrasonication (US), phenolic compounds were complexed with hydrothermally pre-gelatinized rice starch prepared using distilled water or plasma-activated water (PAW). The in vitro digestibility, structural features, rheological and thermal properties, and in vitro antioxidant activity of starch–phenolic complexes were evaluated. The US-assisted starch–MP complex in water had the highest complexing index (CI) value (77.11%) and resistant starch (RS) content (88.35%), resulting in a more compact and stable ordered structure. In all complexes, XRD revealed a new minor crystalline region of V-type, which was stabilized by hydrogen bonding as defined by FTIR and H^1^-NMR. Polyphenols caused a looser gel structure of starch, as imaged by a scanning electron microscope (SEM). Starch–phenolic complexes outperformed other complexes in terms of in vitro antioxidant activity. Gallic acid addition to starch molecules boosted DPPH scavenging activity, notably when synthesized in PAW regardless of US assistance, although having lower CI and RS values than the MP complex. Therefore, this research lays the groundwork for the efficient production of functional food ingredients based on rice starch and polyphenols.

## 1. Introduction

A high-carbohydrate diet is linked to an increased risk of obesity, diabetes, and cardiovascular disease in both developed and developing countries. Carbohydrates, particularly starch, are the primary source of metabolic energy in foods, accounting for approximately 52% of the calories consumed in the US and up to 80% in developing countries. There is a need for strategies to reduce the caloric impact of starchy foods without compromising their sensory properties [1]. Starch complexes with various natural ingredients, particularly phenolic compounds, have been extensively studied for inhibiting starch digestion and lowering glycemic excursions [2]. Slowly digestible starch (SDS) and resistant starch (RS) consumption have been shown to delay the rise in postprandial blood glucose concentrations [3]. However, various physicochemical properties, such as swelling, viscosity, and degree of gelatinization, may be restricted after starch interacts with other guest molecules, altering the overall quality of starchy food products. In order to maintain and improve the nutritional values and quality of starch-based foods, further research into the physicochemical and molecular properties, as well as bioactivity, of starch–phenolic complexes is required. Starch–phenolic conjugates are typically prepared using high amylose starch. Principally, starch can be modified with phenolic compounds to form both inclusive V-type amylose [4] and non-inclusive starch-amylopectin complexes [5]. In both cases, these interactions may reduce starch digestion and lengthen the glycemic response to a given dietary challenge. From this point forward, rice starch may be used to make starch–phenolic conjugates, potentially increasing the utilization of rice starch.

Starch can form inclusion complexes with a variety of small molecules, including alcohols, phenolics, fatty acids, and flavor compounds [6]. Amylose is primarily responsible for complex formation with guest molecules, forming a single left-handed helix stabilized by hydrophobic interactions [7]. The molecular inclusion complex with amylose is determined by the structure of the guest molecules, which can be within the cavity of amylose helices, between the helices, or in both locations [8]. Phenolic compounds are one of the most well-studied guest molecules that form complexes with starch and have anti-glycemic effects because they can inhibit a variety of digestive enzymes (e.g., amylase, protease, and lipase), thereby modifying intestinal glucose transport and release [9,10]. Several studies have reported a significant reduction in starch digestibility when starch and various polyphenols were combined using various physical treatments [11]. Polyphenols can form a V-type crystalline inclusion complex with starch, in which polyphenols are firmly complexed within the cavity of the starch helices [12]. The V–amylose complex is slowly digested and contributes to the development of RS, also known as “RS5” [13]. Non-covalent interactions like hydrogen bonds, hydrophobic interactions, and van der Waals forces can certainly assist polyhydroxy polyphenols in remaining in the starch cavity [14]. According to recent research, dietary phenolics can directly react with starch granules to decrease the digestibility and rheological aspects of foods containing gelatinized starch [15]. Starch–polyphenol complex formation may contribute to changes in the physicochemical and gelling properties of starch [16]. The conditions under which the starch is processed, the variety of starch and phenolics, and the concentration of phenolics, all affect the chemical and techno-functional aspects of modified starch [17]. In order to enhance the quality of starchy foods, it is therefore important to have a better understanding of the interactions between starch and phenolics. The methods of preparation may also alter the interactions between starch and phenolics by influencing the molecular inclusion of the guest molecule to the amylose helix, resulting in a different degree of resistance to starch digestion [11]. Further research could result in the development of a novel method for preparing starch–phenolic complexes that are more efficient in reducing starch digestibility, resulting in a low glycemic response while retaining desirable functional properties.

To date, several methods for producing starch polyphenol complexes have been developed, which can be classified as traditional preparation methods and thermo-mechanical approaches [18]. High hydrostatic pressure [19], high-speed sheering/high pressure homogenization [16], and plasma processing [11] have all emerged as new methods for preparing starch–phenolic complexes. Dual modification methods, such as microwave/ultrasound [16], plasma/pre-gelatinization [11], and pre-gelatinization/high pressure homogenization [20], were also used to increase the amount of phenolics bound to starch and thus decrease starch digestibility more efficiently than pre-gelatinization. The combination appeared to enhance the complexation of starch and polyphenols [11]. Furthermore, novel technologies in starch modification with phenolics could be investigated in order to reduce starch digestibility and easily control the formation of starch complexes. Gao et al. [11] investigated the use of cold plasma discharge and pre-gelatinization in the enhancement of starch–phenolic complexes. During cold plasma treatment, the free radicals produced by gas ionization react physically or chemically with starch, resulting in cross-linking or decomposition and modification of the starch. Cold plasma can cause an increase in surface energy, the addition of functional groups, cross-linking, depolymerization, and a change in the hydrophilic nature of starch, resulting in starch modification [21]. These interactions cause etching, fissures, and pores on the surface of starch granules, as well as changes in the starch’s multi-scale structures [22]. Based on the background and studies mentioned previously, using more than one method improves the starch–phenolic complexes, and novel technologies are still required.

The current study focused on developing a novel strategy for improving starch–phenolic complexes by employing novel techniques, including plasma-activated water (PAW), ultrasonication (US), and their combination, with hydrothermal treatment and then determining the physicochemical and biological properties of the modified starches. Due to the assumption of greater diffusibility in the starch granule, PAW was used in this experiment instead of cold plasma [23]. In order to gain a better understanding, the effects of various polyphenols (gallic acid, sinapic acid, and crude Mon-pu (*Glochidion wallichianum* Muell Arg) leaf extract, MP) on the extent of complexation, physicochemical properties, and in vitro antioxidant activity of resulting starch–phenolic conjugates were studied using a variety of analytical techniques. This study’s choice of MP was made due to its strong antioxidant effects and high phenolic content [24,25]. Overall, this research establishes a scientific framework for producing rice starch–polyphenol conjugates for use in rice starch-based functional foods by emphasizing the contributions of phenolic types, PAW, and US.

## 2. Materials and Methods

### 2.1. Rice Starch Preparation

Wet milling was used to prepare rice flour from Noui Khuea (NK) brown rice (*Oryza sativa* L.), an indigenous underutilized hard rice of Southern Thailand. To obtain flour, NK brown rice (10 kg) was ground in a double-disk stone mill after being soaked in water at a 1:4 (*w*/*v*) ratio for 6 h at 4 °C. After that, the slurry was put in a large fabric bag and compressed using a hydraulic press for 10 min. The moisture level of the wet-milled flour was then brought down to about 11% by drying it in a hot-air oven at 60 °C. Using an MK 5087M Panasonic food processor (Petaling Jaya, Selangor Darul Ehsan, Malaysia), the dried sample was crushed into a powder form and sieved through a 100-mesh sieve. Rice flour was stored at −20 °C in ethylene–vinyl alcohol copolymer (EVOH) bags until used. The storage period was limited to one month.

In order to extract rice starch, rice powder was mixed in a 1:10 (*w*/*v*) ratio with 0.3% sodium hydroxide, stirred for 30 min, and allowed to settle overnight at room temperature (27–29 °C). The precipitate was rinsed with distilled water after the cloudy supernatant was carefully drained. Three repeats of this washing process were conducted in order to make the supernatant clear. The precipitate was resuspended in distilled water and passed through a steel sieve with a mesh size of 100. The resulting filtrate (starch) was air-dried for 12 h at 40 °C [25,26].

### 2.2. Preparation of Plasma-Activated Water (PAW)

The PAW was prepared using air as the working gas, as described by Chaijan et al. [23]. PAW with H_2_O_2_ of 100 ppm (MQuant^®^ Peroxide Test strips, Merck, Darmstadt, Germany) was obtained for 60 min of discharge with pH of 3.0. The freshly prepared PAW was used in the following experiment.

### 2.3. Extraction of Crude Mon-Pu (Glochidion wallichianum) (MP) Leaf Extract

Dried Mon-pu leaf powder (2 g; moisture content < 10%; 25-mesh) was added with 50 mL of distilled water. The mixture was continuously stirred at 50 °C for 15 h. The mixture was filtered using Whatman No.4 filter paper (Whatman International Limited, Kent, UK). The filtrate was freeze-dried, and the powder, referred to as MP, was vacuum packed and stored at −80 °C until used. The total extractable phenolic content in the MP was determined following the Folin–Ciocalteu colorimetric method, using a standard curve of gallic acid (0–100 ppm) [27]. The R^2^ of the standard curve was 0.9993. The phenolic content was expressed as milligrams of gallic acid equivalents (GAEs) per 100 g of MP.

### 2.4. Production of Rice Starch–Phenolic Complex

The starch–phenolic complexes were prepared from the mixture of each phenolic and starch according to the method explained by Zhang et al. [28]. In a nutshell, starch (5 g) was continuously mixed with distilled water or PAW (95 mL) for 20 min. For an additional 30 min, the sample was gelatinized in a boiling water bath (100 °C). Following that, a dropwise addition (50 mL) of 2% (*w*/*v*) gallic acid (Sigma-Aldrich Co., St. Louis, MO, USA), sinapic acid (Sigma-Aldrich Co., St. Louis, MO, USA), or MP solution in the pre-gelatinized starch solution was performed over a 10 min period. The mixtures were incubated at 70 °C for 2 h with a magnetic stirrer to fully mix the starch and phenolic compound. The paste was cooled to 60 °C before being subjected to ultrasonically for 0 or 30 min with interval pulses of 4 s and an amplitude of 20% (Model VCX600, Sonics & Materials, Inc., Newtown, CT, USA) in an ice bath. The mixture was cooled to 25 °C. The mixture was then incubated at 4 °C for 48 h before being centrifuged at 3000× *g* for 10 min. The precipitate was washed three times with absolute ethanol and dried for 24 h at 40 °C. The native starch was used to compare.

### 2.5. Determination of Complexing Index (CI)

The CI value was determined according to the method of Tang and Copeland [29]. In brief, 2.0 g of sample was dispersed in 20 mL of distilled water and then heated at 95 °C for 30 min. Following that, 5.0 g of starch pastes were mixed with distilled water (25 mL) for 120 s. The samples were centrifuged at 4500× *g* for 15 min. The supernatant (500 μL) was combined with distilled water (15 mL) and iodine solution (2 mL). The absorbance (Abs) was measured at 690 nm using a UV–VIS spectrophotometer (Shimadzu Co., Ltd., Kyoto, Japan). The CI values were calculated using the following equation:

(1) $$\mathrm{CI}=\left[\frac{{\mathrm{Abs}}_{\mathrm{without}\;\mathrm{phenolics}\;}-{\;\mathrm{Abs}}_{\mathrm{with}\;\mathrm{phenolics}}}{{\mathrm{Abs}}_{\mathrm{without}\;\mathrm{phenolics}}}\;\right]\times 100$$

### 2.6. In Vitro Digestibility

The contents of rapidly digestible starch (RDS), slowly digestible starch (SDS), and resistant starch (RS) were determined using Englyst et al.’s procedure [30]. In a centrifuge tube, the sample (200 mg) was mixed with sodium acetate buffer (15 mL, pH 5.2). The mixture was pre-incubated at 37 °C for 5 min and 5 mL of the enzyme solution (290 U/mg porcine pancreatic α-amylase and 15 U/mL amyloglucosidase) were added. The mixture was enzymatically hydrolyzed at 37 °C with continuous stirring at 160 rpm for 120 min. The sample was taken at 0, 20, and 120 min intervals. After inactivating the enzyme in a boiling water bath, it was centrifuged (3000× *g*/10 min). The glucose content in the supernatant was quantified using the 3,5-dinitrosalicylic acid (DNS) method, and the RDS, SDS, and RS were calculated using the following equations:

(2) $$\mathrm{RDS}\;\left(\%\right)=\left[\frac{G20-\mathrm{FG}}{\mathrm{TS}}\right]\times 0.9\times 100$$

(3) $$\mathrm{SDS}\;\left(\%\right)=\left[\frac{G120-G20}{\mathrm{TS}}\right]\times 0.9\times 100$$

(4) $$\mathrm{RS}\;\left(\%\right)=\left[\frac{\mathrm{TS}-\left(\mathrm{RDS}+\mathrm{SDS}\right)}{\mathrm{TS}}\right]\times 100$$

where G20 and G120 represent the amounts of glucose released after 20 min and 120 min, respectively. FG is the free glucose content of starch, and TS is total starch weight.

### 2.7. Fourier Transform Infrared (FTIR) Spectroscopy, Proton Nuclear Magnetic Resonance (^1^H-NMR) Spectroscopy, and X-ray Diffraction (XRD)

FTIR analysis was conducted using a Bruker Model Vector 33 FTIR spectrometer (Bruker Co., Ettlingen, Germany) [26]. Thirty-two scans with a resolution of 4 cm^−1^ were used to determine the IR absorption in the range of 500–4000 cm^−1^.

For ^1^H-NMR analysis, native starch and starch–phenolic complexes (10 mg) were dissolved in 0.7 mL of deuterated dimethyl sulfoxide (DMSO-d6) and transferred to 5 mm NMR tubes for ^1^H-NMR spectroscopy analysis at 500 MHz with a Varian NMR spectrometer (Bruker Avance^TM^ NEO, Ascend^TM^, Zurich, Switzerland) to record the spectra at 25 °C with a 5 mm CryoProbe^TM^ probe following standard Varian Pulse Sequences. Top Spin NMR software (Bruker, Ascend^TM^, Zyruch, Switzerland) was used to process the NMR spectra [31].

The XRD pattern of the samples was analyzed by an X-ray diffractometer (Empyrean, PA Nalytical, Almelo, The Netherlands) [26].

### 2.8. Scanning Electron Microscopy (SEM)

The starch granules were adhered to a circular aluminum specimen stub with adhesive tape. After being vertically coated with gold–palladium, the samples were examined using a scanning electron microscope (Gemini SEM, Carl Ziess Microscopy, ZEISS, Jena, Germany) at a 5 kV accelerator potential [26].

### 2.9. Thermal Property Analysis

The thermal property of starch–phenolic complexes was evaluated using a differential scanning calorimeter (DSC; Perkin Elmer DSC6000, Waltham, MA, USA). The starch slurry (1% (*w*/*v*), 9 mL) was added to an aluminum pan and allowed to equilibrate for 6 h before analysis. The pan was heated at a rate of 10 °C/min from 5 °C to 250 °C. Measurements were made of the onset temperature (T_o_), peak temperature (T_p_), ending temperature (T_end_), and gelatinization enthalpy (ΔH).

### 2.10. Dynamic Rheology

The viscoelastic properties of starch–phenolic complexes were determined using the rheometer (Thermo Fisher Scientific Inc., Yokohama, Japan). The starch slurries (40% (*w*/*v*), 2 mL) were immediately transferred between the parallel plates of the rheometer and preheated to 90 °C according to a previous method of Thirumdas et al. [22] with some modifications. There was a 1.0 mm gap, 1% strain (within in the predetermined linear regime), and 1.0 rad/s frequency. The readings fell into the range of linear viscoelasticity. The pastes were cooled at a rate of 2.5 °C/min from 90 to 10 °C, and then maintained at 10 °C for 30 min. G′ and G′′ of the samples were monitored.

### 2.11. Water Absorption Capacity (WAC) and Oil Absorption Capacity (OAC)

The sample (2.5 g dry weight basis, dw) was mixed with 20 mL of distilled water or mustard oil and stirred for 30 min at 25 °C to determine WAC and OAC. The slurry was centrifuged for 10 min at 3000× *g*, and the supernatant was decanted. The weight gain was calculated for WAC or OAC [32]. WAC and OAC were both given as *g*/*g*.

### 2.12. Swelling and Solubility Indices

In pre-weighed centrifuge tubes, starch samples (0.2 g, dw) were mixed with 10 mL of distilled water [32]. The starch suspensions were then incubated in a water bath for 30 min at 60, 70, 80, and 90 °C, with vigorous mixing every 5 min. The tubes were centrifuged (5000× *g*/15 min) after the samples were cooled to room temperature. The supernatant was decanted into moisture cans that had been pre-weighed. The swelling index was used to express the weight gain. The solubility index was calculated by dividing the percentage of soluble solid in supernatant by the sample weight. Swelling index and solubility index were both expressed as *g*/*g*.

### 2.13. Free Radical Scavenging Activity

The 1,1-diphenyl-2-picrylhydrazyl (DPPH) radical scavenging activity of samples was determined by measuring the decrease in absorbance at 515 nm. A UV–VIS spectrophotometer (Shimadzu Co., Ltd., Kyoto, Japan) was used to detect absorbance in 1 cm path length cuvette against methanol in aqueous solution (60:40, *v*/*v*). The results were expressed as a percentage of inhibition [33].

### 2.14. Statistical Analysis

All experiments were carried out in triplicate, and data were collected in the form of mean ± standard deviation (SD). A probability value of *p* < 0.05 was considered significant. Data were subjected to analysis of variance (ANOVA). Comparison of means was carried out by Duncan’s multiple range test. The SPSS for Windows 17.0 software (SPSS Inc., Chicago, IL, USA) was used.

## 3. Results and Discussion

### 3.1. CI and In Vitro Digestibility of Starch–Phenolic Complexes

**Figure 1:**
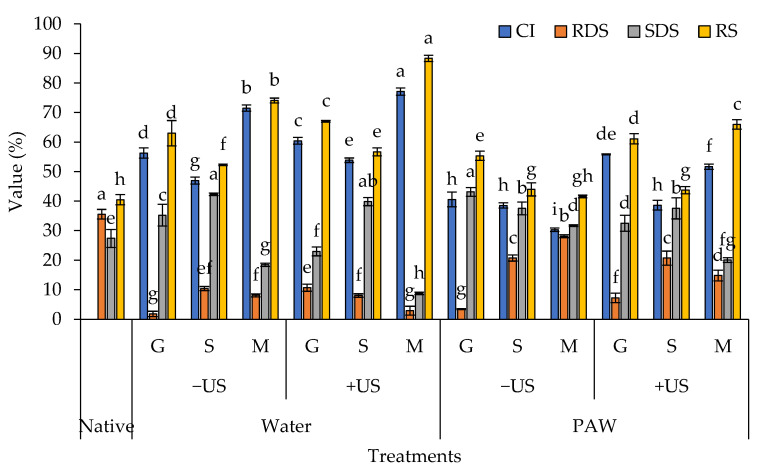
Complexing index (CI), rapidly digestible starch (RDS), slowly digestible starch (SDS), and resistant starch (RS) of rice starch–phenolic complexes. Different phenolic compounds—gallic acid (G), sinapic acid (S), and crude Mon-pu (*Glochidion wallichianum* Muell Arg) (MP) extract—were complexed with hydrothermally pre-gelatinized rice starch prepared using distilled water (Water) or plasma-activated water (PAW) without (−US) and with (+US) the aid of ultrasonication. The native starch (Native) was used to compare. Bars represent the standard deviations from triplicate determinations. Different letters on the bars indicate the significant differences (*p* < 0.05).

Polyphenols have been shown to reduce starch hydrolysis rates in humans by inhibiting digestive enzymes that interact non-covalently with starch. Figure 1 shows the inclusion efficacy of three phenolic compounds, namely gallic acid, sinapic acid, and MP, as represented by the CI values of the resulting complexes. By using US, MP appeared to have a stronger complexation affinity for rice starch in water than gallic and sinapic acids, with a CI value of 77.11% (*p* < 0.05). This could be due to the fact that, when amylose molecules were helped by US, a variety of phenolic compounds with varied degrees of hydrophilicity may interact with them more favorably than pure gallic acid and sinapic acid. According to Li et al. [15], increasing the hydrophobicity of phenolic compounds can increase their affinities for starch granules. The hydrophobic cavity of the amylose molecule may have a low affinity for the incorporation of more polar gallic and sinapic acids into its helical structure, limiting how both phenolic acids interact with starch granules. As a result, pure gallic acid and sinapic acid may struggle to form a link between starch structures [34] or may be unable to trap in the hydrophobic cavity of an amylose helix. Gallic and sinapic acids, on the other hand, are high in hydroxyl groups and may form hydrogen bonds with amylose and amylopectin without forming complexes. Principally, amylopectin has a high degree of polymerization, a complex structure, and a large number of side chains, which allows it to form hydrogen bonds and conjugated bonds with phenolic acid [31]. The structural modification of starch granules by phenolic compounds was critical for converting a small fraction of amylopectin into amylose-like structures, which changed the digestibility of the resulting starch. When compared to the native starch, the RDS and SDS of all complexes were generally altered, increasing the RS content (*p* < 0.05) (Figure 1). It was discovered that the CI value was substantially correlated with the RS content, with the MP treatment having the greatest RS content, followed by gallic acid and sinapic acid (*p* < 0.05), particularly when water was used as a medium with the aid of US (Figure 1). The combination of US and hydrothermal treatments had the highest CI and RS values when compared to other produced methods, indicating greater efficiency in complex formation. A US treatment can typically fragment or disrupt the surface of a solid matrix, such as a starch granule, facilitating amylose leaching and migration and allowing guest molecules to interact with them under heat conditions [16]. All of these factors contribute to the importance of ultrasonic-assisted hydrothermal treatment in achieving successful “green” chemistry in starch–phenolic complexation. No matter the US, the samples treated with PAW had lower CI and RS concentrations than the samples treated with water. This was because the oxidation of the hydroxyl groups induced by PAW may have interfered with the formation of complexes. However, when US was used, the CI and RS concentrations of the PAW-treated samples tended to rise, demonstrating the effectiveness of the method in encouraging the formation of starch–phenolic complexes.

Indeed, the inclusion of phenolics increased the RS and decreased starch digestibility by reducing the activities of digestive enzymes. A V-type amylose inclusion complex is produced by non-covalent interactions between starch and polyphenols that may evade digestion in the small intestine and prevent or treat diabetes and obesity. Meanwhile, starch granules may be employed as an encapsulated delivery matrix to keep polyphenols stable throughout the manufacturing and digestion processes if polyphenols can be kept there by interacting with starch molecules. The digestibility of the starch–polyphenol combination may affect polyphenol release in the human digestive system, thereby improving polyphenol bioaccessibility and health benefits. Most polyphenols with phenolic hydroxyl groups are likely to form hydrogen bonds, hydrophobic interactions, and electrostatic or ionic interactions with starch molecules, resulting in changes in starch digestibility and subsequently affecting starch nutritional properties and phenolic compound release [35]. Phenolic compounds have been shown to reduce starch digestibility through a variety of mechanisms, including decreasing enzyme activity and/or increasing starch ordering [14]. However, phenolic substances have one or more hydroxyl groups that can interact with starch both covalently and non-covalently to produce starch–phenolic complexes, influencing starch structure and digestibility.

### 3.2. FTIR Spectra

**Figure 2:**
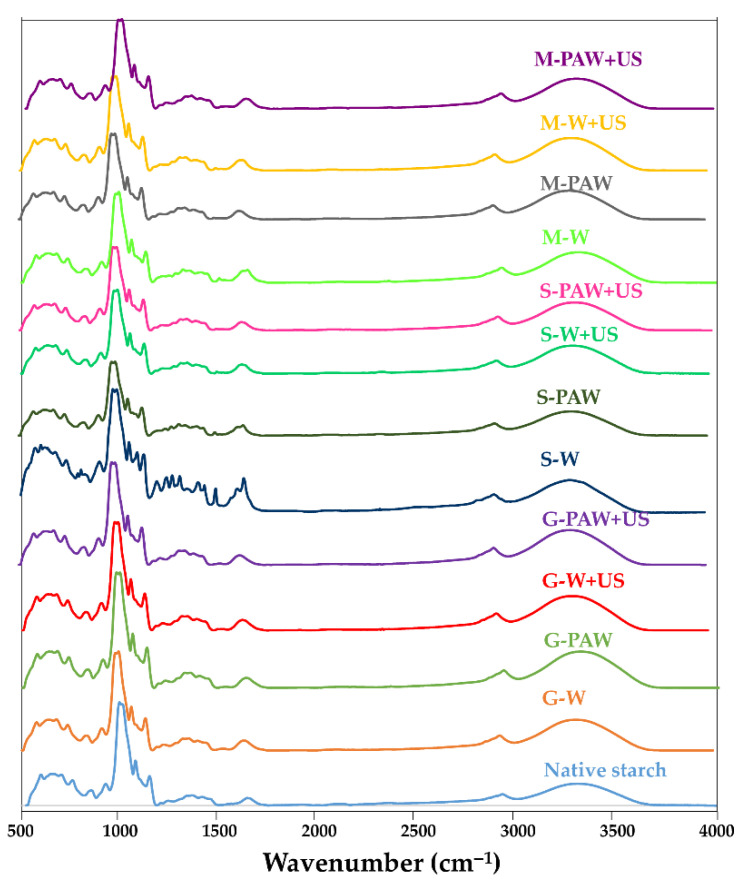
FTIR spectra of rice starch–phenolic complexes. Different phenolic compounds—gallic acid (G), sinapic acid (S), and crude Mon-pu (*Glochidion wallichianum* Muell Arg) (M) extract—were complexed with hydrothermally pre-gelatinized rice starch prepared using distilled water (W) or plasma-activated water (PAW) without and with the aid of ultrasonication (+US). The native starch was used to compare.

Figure 2 depicts the FTIR spectra of native starch and polyphenols–starch complexes. Complexes with gallic acid, sinapic acid, and MP all showed the typical phenolic peaks with a benzene ring OH stretching at 3200–3500 cm^−1^. The presence of intense peaks adjacent at 1450–1600 cm^−1^ was attributed to benzene ring bond stretching, while the bands near 1200–1300 cm^−1^ were caused by C-O/C-C stretching vibrations [36]. The absorption bands in the FTIR spectrum of native starch were prominent at 3298, 2922, 1640, 1363, 1242, 1148, 1078, and 997 cm^−1^. The stretching of aromatic rings caused FTIR absorption bands to appear at 3313, 2925, 1641, 1539, 1410, 1365, 1240, 1148, 1077, 1014, and 664 cm^−1^ for the starch–phenolic complexes. Interestingly, new signals appeared at 1685 and 1447 cm^−1^, indicating the presence of a phenolic compound–starch interaction [31]. Compared to the corresponding mixture, the bands of the starch–sinapic complex were located at 3315, 2925, 1658, 1516, 1457, 1424, 1366, 1335, 1297, 1149, 1077, and 996 cm^−1^. Starch–MP complexes exhibited absorption bands at 3314, 2926, 1641, 1516, 1411, 1365, 1240, 1148, 1077, 1013, 1077, 1013, 997, and 930 cm^−1^. Peaks at 2925 cm^−1^ and 1654 cm^−1^ showed the stretching vibrations of C-H and carbonyl groups, respectively. The peaks at 1601, 1515, and 1471 cm^−1^ were attributed to the stretching vibration of the aromatic rings, while the anhydroglucose ring C-O stretch of C-O-H had a band at 1082 cm^−1^ [11]. The FTIR absorbance at 1047 and 1022 cm^−1^ has been shown to be sensitive to the crystalline/ordered and amorphous structures of the starch surface, respectively. The FTIR spectra of gallic acid and sinapic acid revealed C=O stretching vibrations at 1697 cm^−1^, and additional strong signals at 3276 cm^−1^ and 3520 cm^−1^, which were assigned to C-H stretching vibration on unsaturated carbon and phenolic hydroxyl stretching vibrations, respectively. FTIR spectra of gallic acid and starch appeared in the mixture of gallic acid and starch; the C-O stretching vibration of the anhydro-glucose moiety at 1047 cm^−1^, 1081 cm^−1^, and 1022 cm^−1^, and additional strong signals at 3320 cm^−1^ and 2932 cm^−1^ that were assigned to O-H and C-H stretching vibrations [37]. The deconvoluted spectrum in the range of 800–1200 cm^−1^ was acquired in order to distinguish the variations in the molecular order structure of the native and treated starch surfaces [35]. In conclusion, the variances in FTIR spectra suggested that there may be changes in the primary interactions between various polyphenols and native starch.

### 3.3. ^1^H-NMR Spectroscopy

**Figure 3:**
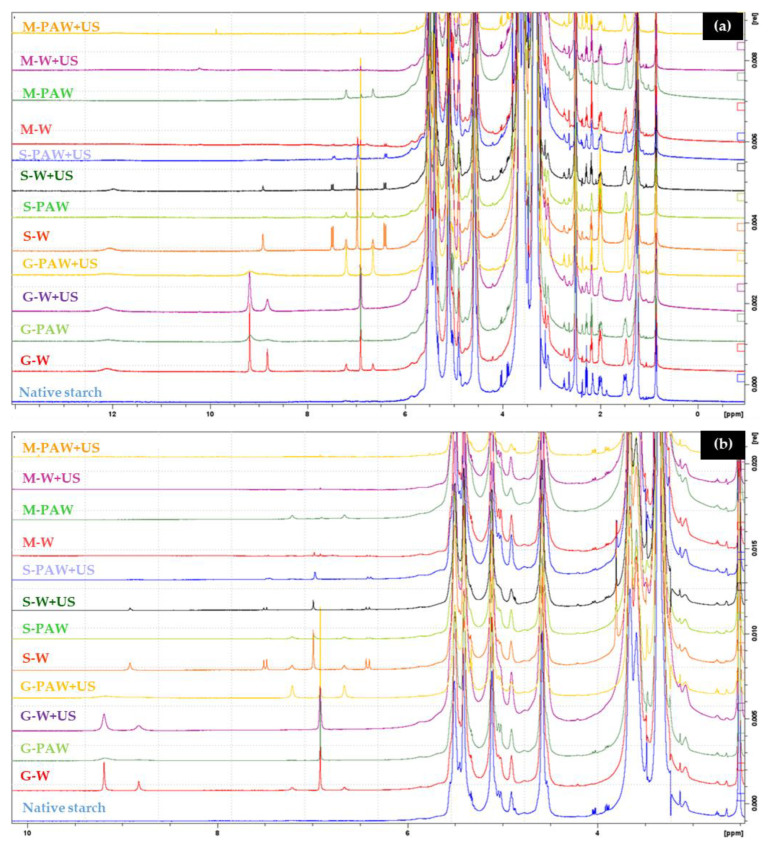
Representative ^1^H NMR spectra of rice starch–phenolic complexes at 0–12 ppm (**a**) and 3–10 ppm (**b**). Different phenolic compounds—gallic acid (G), sinapic acid (S), and crude Mon-pu (*Glochidion wallichianum* Muell Arg) (M) extract—were complexed with hydrothermally pre-gelatinized rice starch prepared using distilled water (W) or plasma-activated water (PAW) without and with the aid of ultrasonication (+US). The native starch was used to compare.

Since starch contains a variety of hydroxyl groups, labile protons can be found as broad signal peaks in ^1^H-NMR spectra, potentially masking the peaks of other functional groups. However, employing DMSO-d6 as the solvent will result in an excellent separation of the signal peaks in the ^1^H-NMR spectrum of starch. As a result, ^1^H-NMR spectroscopy can be used to investigate the interaction between starch and phenolics (gallic acid, sinapic acid, and MP) at 0–12 ppm (Figure 3a) and 3–10 ppm (Figure 3b). The signal peaks at around 6.0–7.5 and 9.0–9.5 ppm correspond to the benzene ring and phenolic hydroxyl groups found in phenolic compounds, respectively. The presence of those characteristic peaks indicated the existence of gallic acid, sinapic acid, and MP in the complexes. In addition, the carboxyl groups of phenolic acid are represented by the signal peak at 12.1 ppm [38]. The terminal groups and hydroxyl protons of starch monomers are responsible for the most noticeable signal peaks in the 4.4–5.6 ppm range of the ^1^H-NMR spectra of the gelatinized–retrograded starch phenolic complex. Additionally, the internal α-1,4 and α-1,6 bonds are represented by the terminal group protons marked as 1 and 1′, respectively [31]. The hydroxyl peaks of the starch–phenolic complex were significantly higher in the range of 4.4–5.6 ppm when compared to the signal peaks of native starch, indicating that phenolic acid interacts with starch molecules via hydrogen bonds and hence combines more readily with amylopectin than with amylose. The hydroxyl peak in the starch–phenolic complex, on the other hand, did not change significantly, which could be due to the low phenolic inclusion rate. The phenolic molecule contains one carboxyl group and two hydroxyl groups, all of which serve as hydrogen bond interaction binding sites. Furthermore, the CH-π bond was the primary interaction for the incorporation of phenolic compounds with starch, i.e., weak CH-π bonds between aromatic residues and starch pyranose rings formed the binding of phenol and starch [39]. The electronic strength of the aromatic ring of phenols is strongly related to the strength of the CH-π bond, whereas the number of phenolic hydroxyl groups decreases the electron intensity, but this weak CH-π bond is readily disintegrated in a low polar solvent system [40].

### 3.4. XRD Pattern

**Figure 4:**
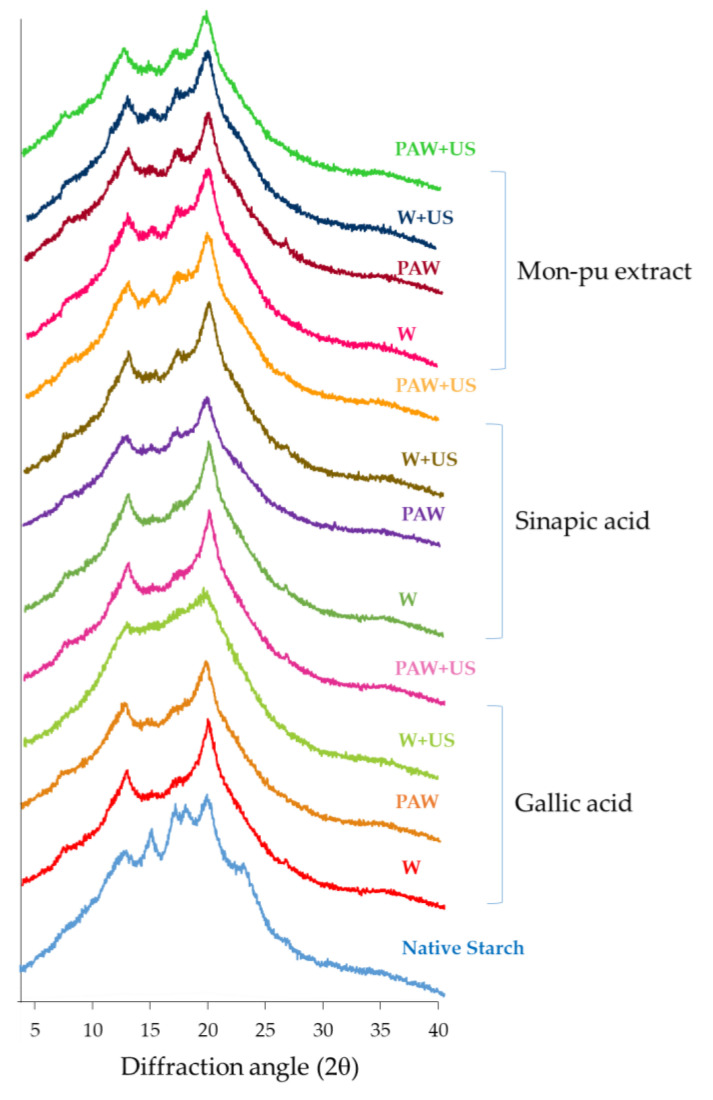
X-ray diffraction spectra of rice starch–phenolic complexes. Different phenolic compounds—gallic acid, sinapic acid, and crude Mon-pu (*Glochidion wallichianum* Muell Arg) extract—were complexed with hydrothermally pre-gelatinized rice starch prepared using distilled water (W) or plasma-activated water (PAW) without and with the aid of ultrasonication (+US). The native starch was used to compare.

The X-ray diffraction spectra of native starch and starch–phenolic complexes are shown in Figure 4. Native starch typically showed an A-type diffraction pattern with specific peaks at diffraction angles (2θ) of 12.86, 15.11, 17.13, 18.07, 19.86, and 22.89. When the starch–phenolic inclusion was formed, the crystalline structure of the starch changed from A-type to V-type with non-covalent interactions within the helical cavity acting as the driving force [16]. The V-type structure had three strong reflections (2θ) at 7.58, 12.86, and 19.88 for the starch complexes with gallic acid, sinapic acid, and MP, respectively, as shown in Figure 3 [16,41]. Non-covalent interactions, such as CH-π interactions along the α-(1→4) glycosidic bond and hydrogen bond, were primarily associated with amylose–polyphenol self-assembly, which increased RS levels in the resulting inclusion [41]. This outcome agreed with the conclusions made by Gutiérrez and Tovar [13], who found high-amylose corn starch–gallic acid inclusions as V-type complexes. In the presence of a guest molecule, amylose undergoes structural changes with hydrophobic cavities that attract the guest molecules [42] and produces V-type complexes [43]. As a result, phenolic compounds may form interaction with the rice starch to form V-type inclusion complexes driven by non-covalent interactions [12]. Typically, the amorphous regions of starch molecules are most affected by physical techniques during starch modification when compared to crystalline regions. The variety of the non-covalent interactions between starch and phenolics modifies the characteristics of the starch; these linkages include hydrogen bonds, hydrophobic interactions, electrostatic interactions, and ionic interactions [12]. The interaction of starch and phenolic substance can result into two types of complexes: V-type and non-V-type complexes [12]. Commonly, amylose single helixes and hydrophobic contacts are responsible for the creation of V-type inclusion complexes [44]. Small guest molecules, such as gallic acid, are strongly complexed inside the space of amylose helices to form inclusion complexes because the cavity has a hydrophobic atmosphere [37]. The X-ray diffraction pattern differs because inclusion complexes have a distinct crystal structure (V-type) compared to native starch (A-type). Non-V-type inclusion complexes have a looser structure than V-type inclusion complexes, but there is no change in the crystallinity of starch. Strong phenolics–starch interactions in V-type inclusion complexes are more tolerant of enzymatic digestion than non-V-type intercalation [45].

### 3.5. Morphological Structure

**Figure 5:**
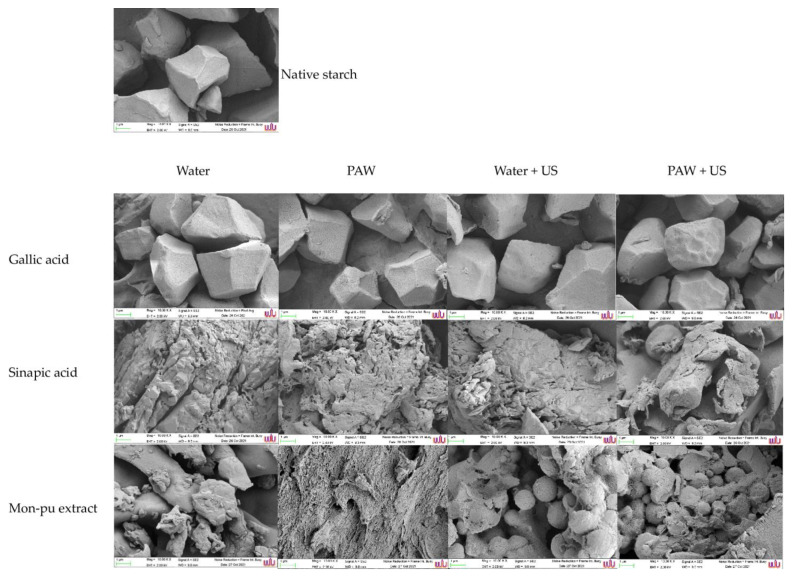
Scanning electron microscopic (SEM) images of rice starch–phenolic complexes. Different phenolic compounds—gallic acid, sinapic acid, and crude Mon-pu (*Glochidion wallichianum* Muell Arg) extract—were complexed with hydrothermally pre-gelatinized rice starch prepared using distilled water (Water) or plasma-activated water (PAW) without and with the aid of ultrasonication (+US). The native starch was used to compare. EHT: 2.0 kV. Magnification: 10,000×.

The effects of different polyphenols (gallic acid, sinapic acid, and MP) on the surface morphology of the starch–phenolic complex were studied using SEM (Figure 5). The native starch granules were polygonal, irregular in shape, and had a smooth surface. After complexation, the starch granules’ natural structure was totally disrupted, and during gelatinization, agglomerates formed (Figure 5). In comparison to native starch, the starch–polyphenol complexes typically formed a porous gel matrix with a looser structure. This could be explained by the breakdown of granular structure and the emergence of relatively short linear amylose chains, which later accumulated and evolved into amorphous particles [46]. The existence of layered strips, which could have been caused by degradation after various processing methods, set apart the surface topography. This finding was consistent with Zhu et al. [47], who found that including phenolic compounds from pomegranate peel resulted in a looser gel matrix of the resulting wheat starch–phenolic complex. The interaction of wheat starch and lotus leaf flavonoids had similar outcomes [48]. The morphological structure of the ultrasonically treated starch–phenolic complexes was different, with a higher amount of flaky and groove forms, deeper layered sheets, and an irregular granule surface. A combination of hydrothermal and ultrasonically treated starch–phenolic complexes may form disrupted granules due to severe cavitation force.

### 3.6. Thermal Properties

Table 1 depicts the thermal properties of various starch–phenolic complexes as measured by DSC. The incorporation of each phenolic compound into starch molecules increased the onset temperature (T_o_), melting temperature (T_p_), end temperature (T_end_), and enthalpy (ΔH) compared to the native starch (*p* < 0.05), indicating the formation of a more ordered structure. Starch–gallic complexes had the highest gelatinization temperature and energy (T_p_ and ΔH) among the phenolics used, implying a denser molecular structure of the resulting complex. These findings are most likely explained by phenolic acid interacting with the starch–water matrix during complex formation, resulting in a shift in gelatinization temperature [49]. Gallic acid, in particular, formed a hydrogen bond with the starch chain, which contains hydroxyl and carboxyl groups. The weak interaction of starch–gallic conjugation, on the other hand, is due to limited hydroxyl groups and small size of gallic acid. As a result, a portion of the gallic acid in the unstable complexes may attach to the starch crystal surface and initiate thermal degradation at high temperatures [50]. This phenomenon may reduce water mobility and availability for the gelatinization process, which could explain why gelatinization occurs to a lesser extent in the presence of phenolic acid by having higher ΔH values [38]. Phenolic acids can also typically interact with amylose and amylopectin via hydrogen bonds or van der Waals interactions, increasing the binding forces between crystallites and amorphous matrices to varying degrees [51]. This may necessitate more heat energy for gelatinization, as evidenced by an increase in melting stability, which corresponded to a decrease in the swelling power of gallic acid, sinapic acid, and MP-containing starch. Similar findings have been reported elsewhere in relation to the endotherms of starch gelatinization (temperatures and ΔH) in the presence of phenolic compounds [49].

When compared to hydrothermal alone, the use of PAW- and US-assisted processes for complex formations resulted in a lower gelatinization temperature while increasing the ΔH with a border melting peak (Table 1). These results reveal that the complex formation method had an impact on the molecular interaction and structural rearrangement of starch and phenolic compound. All thermal parameters were found to be the lowest in starch–phenolic complexes produced by PAW–hydrothermal–US treatment due to excessive chemical and physical forces (Table 1), resulting in a less ordered structure and complex formation. Under the influence of external conditions, the starch chain rotates due to intramolecular hydrogen bonds, creating a left-handed helical cavity, which could interact with the present polyphenol via hydrophobic interaction in the helical cavity [41]. A severely generated condition may destroy the starch’s helical structure, resulting in lower starch inclusion complex formation with polyphenols. Interestingly, when compared to other produced methods with the same incorporated phenolics, the combination of PAW and hydrothermal resulted in samples with the highest ΔH (Table 1). It was unclear, but the intact interaction of oxidized starch with polyphenols could account for it. This led to the development of a compact crystalline structure with a high melting energy requirement. However, there was a slight difference in T_o_, T_p_, and T_end_ between the PAW–hydrothermal-made complex and the complex prepared using water as a medium with the aid of US (Table 1).

### 3.7. Dynamic Rheology

**Figure 6:**
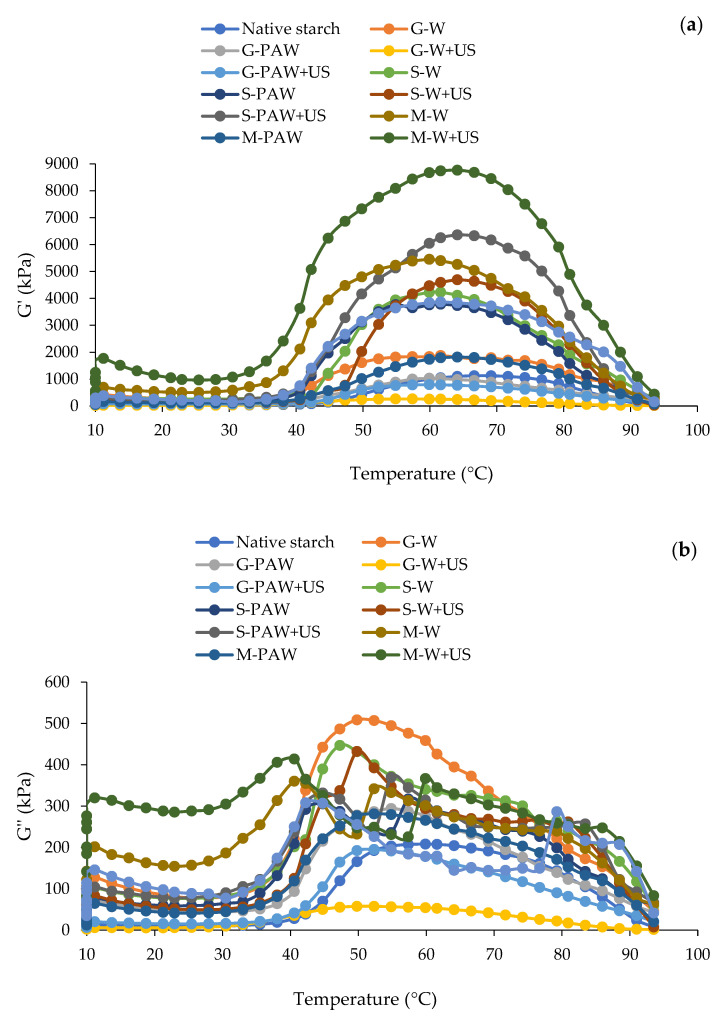
Storage modulus (G′) (**a**) and loss modulus (G″) (**b**) of rice starch–phenolic complexes. Different phenolic compounds—gallic acid (G), sinapic acid (S), and crude Mon-pu (*Glochidion wallichianum* Muell Arg) extract (M)—were complexed with hydrothermally pre-gelatinized rice starch prepared using distilled water (W) or plasma-activated water (PAW) without and with the aid of ultrasonication (+US). The native starch was used to compare.

The rheological properties of starch–phenolic complexes produced by three phenolic and various methods, are displayed in Figure 6. The inclusion of phenolic compounds increased or decreased the maximum G′ and G″ during heating at temperatures ranging from 10 to 90 °C, depending on the type of phenolic and produced methods used. The complexes containing MP and gallic acid showed the highest and lowest G′, respectively (Figure 6). The undegraded complexes of MP with starch could account for the high G′ and G″. It should be noted that all US-produced complexes had lower G′ and G″ values than other methods of production. This was caused by the intense cavitation effect, which resulted in the molecular disruption of starch granules. The creation of a three-dimensional gel network was caused by the fractured starch granules and the leakage of amylose strands from the granules [52]. When the starch granules swell, phenolic acid can help them create composite networks. The phenolic acid may be able to maintain partially disintegrating starch granules, with some of them filling in the crevices and voids between the swelling granules, despite the difference in particle sizes between the two (up to several times the volume of the original granules). Further heating led to prolonged granule rupture and starch degradation, complete melting of the crystallites, and loosening of inter-chain connections [53]. The incorporation of phenolic acid into starch resulted in an increased G′, toward 90 °C. Although most phenolic acids are crystalline at 90 °C, they are not involved in matrix formation via hydrogen bonding. As a result, phenolic acid may dilute the melted starch matrix, significantly improving structural breakdown [17]. This could be due to the realignment and reassociation of starch chains during gelling. There was increase in G′ and decrease in G″ after holding for 30 min at 10 °C (Figure 6). The interactions between broken chains, newly generated function groups (carboxyl), or crosslinking with water may account for the observed modulus alterations. The high strength of the starch gel is influenced by a higher G′. As a result of this, the decrease in G′ after ultrasonication results in the formation of weak gels. During cooling, the amylose molecules formed hydrogen connections with one another, expanding the gel network and raising G′. Wang et al. [54] reported that the structure of amylopectin is more essential than the structure of amylose in influencing rheological properties. The use of intermediate starch in this study confirmed the previous statement by dramatically increasing G′ and G″ after polyphenol complexation. The addition of ultrasonication caused significant oxidation, crosslinking, and molecular disruption of starch and polyphenols, altering the rheological properties of the starch. This was consistent with the complexation of quercetin, rutin, ferulic acid, and gallic acid with starch [11,14,17,49], indicating that phenolic molecules created a robust cross-linkage and improved the viscoelasticity of starch. The limited starch granule swelling could also significantly increase the stiffness of the amylose gel [11]. The higher tan δ value of starch–phenolic complexes suggested that the viscous component was obtained following complexation. This could be because the interaction between phenolic compounds and starch hampered network formation or restricted amylose leaching, resulting in a weaker gel [55]. However, the proposed technique appeared to influence the rheological behavior of starch–phenolic complexes, with ultrasonically produced starch–gallic complexes having the lowest G′ and G″ (Figure 6).

### 3.8. WAC and OAC

**Figure 7:**
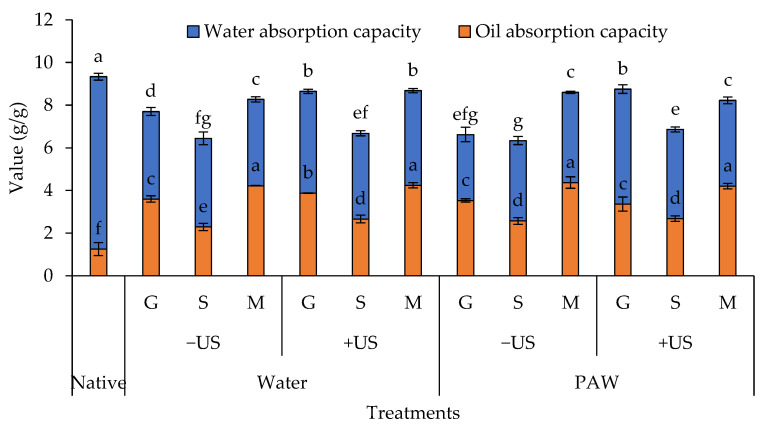
Water absorption capacity (WAC) and oil absorption capacity (OAC) of rice starch–phenolic complexes. Different phenolic compounds—gallic acid (G), sinapic acid (S), and crude Mon-pu (*Glochidion wallichianum* Muell Arg) (M) extract—were complexed with hydrothermally pre-gelatinized rice starch prepared using distilled water (Water) or plasma-activated water (PAW) without (−US) and with (+US) the aid of ultrasonication. The native starch (Native) was used to compare. Bars represent the standard deviations from triplicate determinations. Different letters on the bars within the same parameter indicate the significant differences (*p* < 0.05).

The WAC and OAC of the untreated and phenolic-treated starches are shown in Figure 7. When compared to native starch (9.33 *g*/*g*), the WAC of all starch–phenolic complexes was significantly reduced to 6.33–8.68 *g*/*g*. Due to the fact that the hydrogen bond was primarily responsible for the interaction between starch and phenolic compound, the inability of the starch moiety to interact with water may explain the low WAC of the resulting starch–phenolic complexes. Starch–MP complexes had the highest WAC among the phenolic-treated samples, followed by gallic complexes and sinapic complexes (*p* < 0.05). This indicated that the type of phenolic substance used had an impact on the WAC of the resulting complexes. The numerous phenolic constituents in MP with varying degrees of hydrophobicity may interact with starch molecules rather than hydrophilic gallic acid and sinapic acid. It should be noted that the PAW-treated starch, before conjugating with each phenolic compound by hydrothermal treatment, had the lowest WAC (*p* < 0.05), whereas all samples treated with ultrasound had the largest WAC (Figure 7). The lesser WAC of the PAW-treated samples may relate to the oxidation of the -OH groups in starch molecules as altered by reactive species in PAW, resulting in inter-/intra-molecular crosslinking among starch chains The molecular crosslinking of starch may obtain unavailable OH groups to bind with water, resulting in low WAC in PAW-treated samples. On the other hand, the combination of intense ultrasonication could expose the new free OH group in starch molecules to easily interact with water, providing more affinity to form hydrogen bonds with it.

All starch–phenolic complexes had a significantly higher OAC than native starch (*p* < 0.05), indicating that the formation of starch–phenolic complexes could enhance the interaction with lipids. The highest OAC was observed in all starch–MP complexes (4.22–4.37 *g*/*g*), followed by starch–gallic complexes (3.36–3.87 *g*/*g*) and starch–sinapic complexes (2.29–2.66 *g*/*g*) (*p* < 0.05). This may be related to the large number of hydrophobic polyphenols found in MP that can hydrophobically interact with lipids. According to Li et al. [15], adding hydrophobic phenolic compounds to starch could significantly increase the oil adsorption of the resulting starch–phenolic complex. It should be noted that the produced methods had a negligible effect on OAC as compared with the same phenolic used. Therefore, the type of phenolic compound incorporated was more prominent on the OAC of the starch–phenolic complex than that produced method (Figure 7).

### 3.9. Water Solubility and Swelling Indices

**Figure 8:**
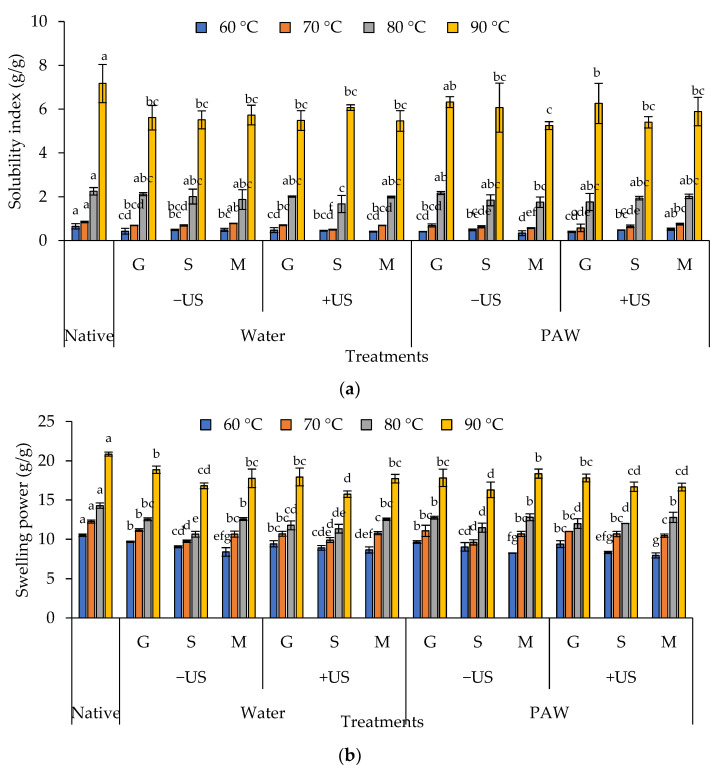
Water solubility index (**a**) and swelling power (**b**) of rice starch–phenolic complexes at different temperatures. Different phenolic compounds—gallic acid (G), sinapic acid (S), and crude Mon-pu (*Glochidion wallichianum* Muell Arg) (M) extract—were complexed with hydrothermally pre-gelatinized rice starch prepared using distilled water (Water) or plasma-activated water (PAW) without (−US) and with (+US) the aid of ultrasonication. The native starch (Native) was used to compare. Bars represent the standard deviations from triplicate determinations. Different letters on the bars indicate the significant differences (*p* < 0.05).

The water solubility index (WSI) and swelling power (SP) of native rice starch and its complex with phenolic compounds are presented in Figure 8. Overall, the complexes appeared to have a lower WSI and SP than the native one (*p* < 0.05). The hydration property of starch–phenolic complexes, as demonstrated by the WSI, supported this observation (Figure 8). Previous research on the reduction in SP in potato and rice starch complexed with gallic acid, caffeic acid, and ferulic acid endorsed our findings [31,34]. The addition of phenolic compounds may compact the crystal structure of starch, resulting in slow amylose leaching and, as a result, preventing gelatinization and decreasing the solubility and swelling of the complexes [15]. There was a slight difference in the WSI and SP between the three phenolic incorporated and produced methods (Figure 8), indicating that both parameters had a lesser impact on the WSI and SP. It should be noted that the WSI and SP increased gradually as the tested temperature increased due to the molecular diffusion of water into starch granules and thermal degradation, which increased soluble component leaching and secondary bond breaking in starch molecules.

### 3.10. Free Radical Scavenging Activity

**Figure 9:**
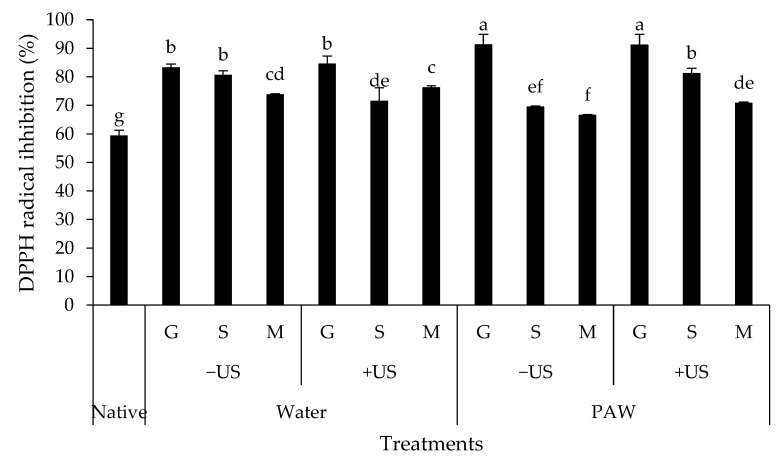
DPPH radical inhibition of rice starch–phenolic complexes. Different phenolic compounds—gallic acid (G), sinapic acid (S), and crude Mon-pu (*Glochidion wallichianum* Muell Arg) (M) extract—were complexed with hydrothermally pre-gelatinized rice starch prepared using distilled water (Water) or plasma-activated water (PAW) without (−US) and with (+US) the aid of ultrasonication. The native starch (Native) was used to compare. Bars represent the standard deviations from triplicate determinations. Different letters on the bars indicate the significant differences (*p* < 0.05).

Figure 9 displays the ability of native starch and starch–phenolic complexes to scavenge DPPH radicals. The presence of hydroxyl groups in starch and phenolic substances corresponds to the radical inhibition of the formed complexes. Depending on the phenolic type employed, the DPPH radical scavenging activity of the starch–phenolic complexes was considerably higher than that of native starch (*p* < 0.05). The higher antioxidant potential of starch–phenolic complexes was caused by an interaction between starch and phenolic compounds, which varied depending on the structure–activity relationships of the complexes [56]. The starch–gallic complex had the highest DPPH radical scavenging activity (91.15%), followed by the starch–sinapic complex (80.56%) and the starch–MP complex (76.18%) (*p* < 0.05). As a result, the phenolic type governed the radical scavenging activity of the starch–phenolic complex. This study supports the findings of Van Hung et al. [57], who found that starch–ferulate complexes with a higher degree of substitution had greater radical scavenging activity than native starch. It should be noted that starch–gallic complexes produced by PAW and PAW plus US had the highest DPPH radical scavenging activity (91.15%), which was 1.54 times that of native starch (Figure 9). Due to the fact that starch molecules acted as an entrapping matrix in the structure of the phenolic compounds, applying high force to PAW and PAW + US caused more available OH groups of starch or polyphenols to be released from the dense matrix, resulting in greater free radical scavenging activity. In conclusion, the radical scavenging activity of the starch–phenolic complex was found to be dependent on phenolic type and preparation method. In products such as muscle foods and lipid-based foods, the complexes can be used as a dual-functional ingredient that serves as a stabilizing agent and an antioxidant [58].

## 4. Conclusions

The CI and in vitro digestibility of the rice starch–phenolic complexes varied according to phenolic type (gallic acid, sinapic acid, and MP) and method of production (hydrothermal treatment in water or PAW with or without US). The starch complexed with MP assisted by ultrasonication at a 20% amplitude for 30 min had the highest CI value (77.11%) and RS content (88.35%). FTIR, ^1^H-NMR, XRD, and DSC analyses revealed that all starch–phenolic complexes formed V-type complexes. In comparison to native starch, the starch–phenolic complexes exhibited increased, DPPH radical scavenging activity, which could be due to the formation of non-inclusive complexes. Overall, the US-assisted hydrothermal treatment produced more complexes than other methods, resulting in the improved molecular inclusion of starch–phenolic complexes and increased in vitro antioxidant activity. This study addressed a novel strategy for improving rice starch–polyphenol inclusions, which may increase interest in the food industry in using novel polyphenol-based starchy functional food ingredients. However, the large-scale manufacturing of the inclusion complex between rice starch and phenolics is required for industrial applications. The release of phenolic compounds from rice starch–phenolic conjugates, as well as the storage stability and usability of the rice starch–phenolic complex generated by this method in food products, will need to be researched further in the future.

## Figures and Tables

**Table 1:** Thermal properties of rice starch–phenolic complexes.

Parameters	Native	Water						PAW					
		−US			+US			−US			+US		
		G	S	M	G	S	M	G	S	M	G	S	M
T_o_ (°C)	52.4 ± 5.2 g	114.2 ± 1.6 de	134.7 ± 2.1 b	117.3 ± 5.0 ef	126.5 ± 3.4 b	111.0 ± 2.1 a	126.3 ± 1.6 b	122.8 ± 0.3 bc	111.6 ± 0.6 f	133.0 ± 0.4 cd	107.8 ± 4.8 bc	118.1 ± 4.6 ef	133.0 ± 0.5 a
T_p_ (°C)	87.9 ± 4.2 h	151.2 ± 1.1 a	150.2 ± 0.1 b	127.3 ± 4.5 f	144.3 ± 0.4 b	130.6 ± 1.9 ef	138.5 ± 1.0 bcd	143.0 ± 5.4 bc	135.9 ± 0.1 de	137.8 ± 4.3 cd	119.4 ± 3.6 g	116.0 ± 5.2 g	139.4 ± 4.7 bcd
T_end_ (°C)	107.9 ± 1.7 f	171.5 ± 3.5 a	161.8 ± 0.6 b	127.8 ± 5.3 d	156.0 ± 0.1 bc	151.5 ± 1.4 cd	150.4 ± 1.6 cd	156.2 ± 5.5 bc	155.3 ± 2.4 cd	148.8 ± 4.9 cd	150.2 ± 4.9 cd	150.4 ± 5.3 e	154.2 ± 2.3 cd
ΔH (J/g)	550.1 ± 8.1 k	996.2 ± 9.8 b	948.8 ± 1.2 c	706.7 ± 4.4 h	849.5 ± 1.0 d	663.9 ± 4.4 i	713.3 ± 2.2 gh	1025.2 ± 5.2 a	616.4 ± 5.8 j	716.0 ± 3.5 g	748.8 ± 5.9 e	620.7 ± 1.8 j	727.7 ± 0.5 f

Different phenolic compounds—gallic acid (G), sinapic acid (S), and crude Mon-pu (*Glochidion wallichianum* Muell Arg) (M) extract—were complexed with hydrothermally pre-gelatinized rice starch prepared using distilled water (Water) or plasma-activated water (PAW) without (−US) and with (+US) the aid of ultrasonication. The native starch (Native) was used to compare. Values are given as mean ± standard deviation (SD) from triplicate determinations. Different letters in the same row indicate the significant differences (*p* < 0.05). T_o_ = onset temperature. T_p_ = Endothermic peak. T_end_ = End temperature. ΔH = Enthalpy.

## Data Availability

The data presented in this study are available in the article.
